# The effects of focal epileptic activity on regional sensory-evoked neurovascular coupling and postictal modulation of bilateral sensory processing

**DOI:** 10.1038/jcbfm.2013.115

**Published:** 2013-07-17

**Authors:** Sam Harris, Michael Bruyns-Haylett, Aneurin Kennerley, Luke Boorman, Paul G Overton, Hongtao Ma, Mingrui Zhao, Theodore H Schwartz, Jason Berwick

**Affiliations:** 1 Department of Psychology, University of Sheffield, Sheffield, UK; 2 Department of Neurological Surgery, Brain and Spine Center, Brain and Mind Research Institute, Weill Cornell Medical College, New York Presbyterian Hospital, New York, New York, USA

**Keywords:** barrel cortex, electrophysiology, epilepsy, intrinsic optical imaging, neurovascular coupling

## Abstract

While it is known that cortical sensory dysfunction may occur in focal neocortical epilepsy, it is unknown whether sensory-evoked neurovascular coupling is also disrupted during epileptiform activity. Addressing this open question may help to elucidate both the effects of focal neocortical epilepsy on sensory responses and the neurovascular characteristics of epileptogenic regions in sensory cortex. We therefore examined bilateral sensory-evoked neurovascular responses before, during, and after 4-aminopyridine (4-AP, 15 mmol/L, 1 *μ*L) induced focal neocortical seizures in right vibrissal cortex of the rat. Stimulation consisted of electrical pulse trains (16 seconds, 5 Hz, 1.2 mA) presented to the mystacial pad. Consequent current-source density neural responses and epileptic activity in both cortices and across laminae were recorded via two 16-channel microelectrodes bilaterally implanted in vibrissal cortices. Concurrent two-dimensional optical imaging spectroscopy was used to produce spatiotemporal maps of total, oxy-, and deoxy-hemoglobin concentration. Compared with control, sensory-evoked neurovascular coupling was altered during ictal activity, but conserved postictally in both ipsilateral and contralateral vibrissal cortices, despite neurovascular responses being significantly reduced in the former, and enhanced in the latter. Our results provide insights into sensory-evoked neurovascular dynamics and coupling in epilepsy, and may have implications for the localization of epileptogenic foci and neighboring eloquent cortex.

## Introduction

Cortical responses to sensory stimuli may be altered in focal neocortical epilepsy.^1, 2^ In occipital lobe epilepsy, regions of visual cortical dysfunction ipsilateral to the epileptogenic region can be identified using functional magnetic resonance imaging (fMRI).^3^ Postictal attenuation of auditory event-related potentials ipsilateral to the epileptogenic focus has also been reported in temporal lobe epilepsy.^4^ Assessment of cortical dysfunction can therefore not only elucidate the relationship between partial onset epilepsy and sensory systems, but also provide important lateralizing information on the seizure onset zone (SOZ). However, little is known on the effects of focal neocortical epilepsy on sensory-evoked neurovascular coupling. Therefore, whether debilitated sensory-evoked neurovascular coupling is also a feature of epileptogenic zones in sensory regions, and whether recurrent seizure activity produces enduring effects on neurovascular responses, both ipsilateral and contralateral to the SOZ, remain unexplored. In nonpathologic brain states, evaluation of sensory-evoked neurovascular responses in anesthetized-rodent models can offer valuable insights into the nature of neurovascular coupling, and guide refinement of mathematical models that enable interpretation of perfusion-based imaging signals in terms of underlying neuronal activity.^5, 6, 7, 8, 9, 10, 11^ Assessment of neural and hemodynamic responses to sensory stimulation before, during, and after epileptiform activity can therefore interrogate the stability of neurovascular coupling mechanisms in epilepsy, which have been recently suggested to be altered in acute models.^12, 13^ This remains an important question, as a detailed understanding of neurovascular coupling is crucial to the correct interpretation of perfusion-related imaging signals in epilepsy, such as blood oxygen level-dependent (BOLD) fMRI, that are increasingly used during presurgical evaluation of intractable epilepsy, and which typically assume linearity in neurovascular coupling.^14^

Using the 4-aminopyridine (4-AP) acute model of focal epilepsy in the rat somatosensory cortex, we hypothesized that the supranormal metabolic stress placed on the neurovascular unit during ictal-like discharges may modulate conventional neurovascular responses to sensory stimulation. Further, we posited that recurrent seizures may have lasting effects on sensory-evoked neurovascular responses not only in the cortex ipsilateral to the SOZ but also in contralateral cortex. We therefore used concurrent multichannel electrophysiology to measure the current source density (CSD) of evoked field potentials (FPs), and two-dimensional optical imaging spectroscopy to obtain spatial maps of oxyhemoglobin (HbO), deoxyhemoglobin (Hbr), and total hemoglobin (Hbt) concentration in the rat neocortex. We subsequently evaluated regional neurovascular responses to vibrissal stimulation during 4-AP-induced focal neocortical seizures and examined bilateral evoked responses after cessation of seizure activity.

## Materials and methods

### Animal Preparation and Surgery

All procedures described were performed with UK Home Office approval under the Animals (Scientific Procedures) Act of 1986. Female hooded Lister rats (total *N*=12, weighing 220 to 400 g) were kept in a 12-hour dark/light cycle environment at a temperature of 22°C, with food and water provided *ad libitum*. Animals were anesthetized with urethane (1.25 g/kg) intraperitoneally, with atropine being administered subcutaneously (0.4 mg/kg) to reduce mucous secretions during surgery. Before surgery and throughout the experiment, desired depth of anesthesia was verified by confirming the absence of a corneal reflex and withdrawal reflex to hindpaw pinch. A homoeothermic blanket (Harvard Apparatus, Edenbridge, UK) and rectal probe were used to monitor and regulate core body temperature at a stable 37°C. Animals were tracheotomized to allow artificial ventilation with pressurized room air and monitoring of end-tidal CO_2_. Blood-gas and end-tidal CO_2_ measurements were used to inform adjustment of ventilator parameters so as to maintain the animal within normal physiologic limits (average values: pO_2_=89.2 mm Hg±8.7, pCO_2_=28.1 mm Hg±5.9). The left femoral artery and vein were cannulated to allow measurement of arterial blood pressure and phenylephrine infusion (0.13 to 0.26 mg/h to maintain normotension between 100 and 110 mm Hg), respectively. Animals were placed in a stereotaxic frame (Kopf Instruments, Tujunga, CA, USA), and the skull overlying stereotaxic coordinates 2 mm anterior to lambda to 3 mm anterior of bregma, and from 1 to 6 mm from midline, thinned to translucency using a dental drill, to expose a large portion of the somatosensory and motor cortices. This was performed either unilaterally (*N*=6) or bilaterally (right and left hemispheres, *N*=6). A circular plastic ‘well' was placed over the cranial window using dental cement and filled with saline to reduce specularities from the brain surface during imaging. In all experiments described, subcutaneous stainless-steel stimulation electrodes (insulated to within 2 mm of the tip) were inserted in a caudal direction into the mystacial pad between rows A/B and C/D so as to ensure electrical stimulation of all vibrissae (either unilaterally or bilaterally).

### Epilepsy Model

The potassium channel blocker 4-AP (Sigma-Aldrich, Gillingham, UK, 15 mmol/L, 1 *μ*L) was used to generate focal seizure-like discharges^12, 13, 15^ in the right vibrissal cortex (RVC). 4-AP was infused at a depth of 1,500 *μ*m via the fluidic port in a multichannel microelectrode (Neuronexus Technologies, Ann Arbor, MI, USA) over a 5-minute period (0.2 *μ*L/min) using a 10-*μ*L Hamilton syringe and syringe pump (World Precision Instruments Inc., Sarasota, FL, USA).

### Two-Dimensional Optical Imaging Spectroscopy

Two-dimensional optical imaging spectroscopy was used to measure changes in cerebral HbO, Hbr, and Hbt concentration. A Dalsa 1M30P camera (Billerica, MA, USA) operating in 4 × 4 binning mode was used to record the images, with each pixel representing ∼75 *μ*m × 75 *μ*m. Spatial maps of cortical hemodynamic responses were generated using a Lamda DG-4 high speed filter changer (Sutter Instrument Company, Novata, CA, USA). Illumination of the cortex was conducted at four different wavelengths specifically selected as two pairs (495±31 nm FWHM (full width at half maximum) and 559±16 nm FWHM; 575±14 nm FWHM and 587±9 nm FWHM), with each pair sharing a similar total absorption coefficient (thus sampling a similar tissue volume) but possessing antipodal HbO and Hbr absorption coefficients (so as to maximize the signal-to-noise ratio). Camera frame rate was set at 32 Hz, and synchronized to the filter switching, resulting in an effective frame rate of 8 Hz per wavelength. Image data were subjected to spectral analysis consisting of a path length scaling algorithm described in detail previously.^16, 17^ Briefly, the algorithm uses a modified Beer–Lambert Law in conjunction with a path-length correction factor for each wavelength used, based on Monte Carlo simulations of light transport through tissue. Baseline concentration of Hbt was estimated at 104 *μ*mol/L^18^ with saturation estimated at 50%. The spectral analysis produced 2D images over time of HbO, Hbr, and Hbt, the latter being proportional to cerebral blood volume (CBV) under the assumption of a constant hematocrit.^5^

### Localization of Vibrissal Somatosensory Cortex for Electrode Implantation

**Figure 1:**
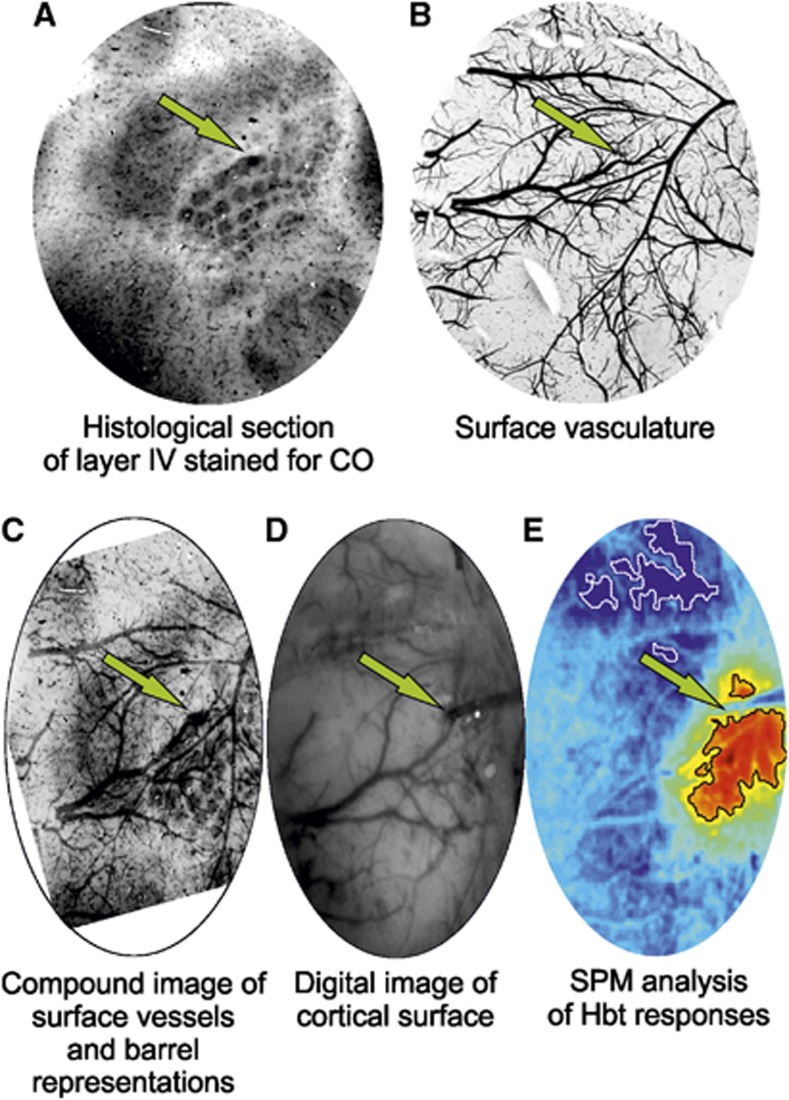
Histologic confirmation of electrode implantation site in a representative subject. Electrode sites are indicated with a green arrow. (**A**) Tangential histologic section of cortical lamina IV showing barrel structures stained for cytochrome oxidase (CO) reactivity and location of electrode lesion in barrel E4. (**B**) Tangential surface section of cortex showing superficial vasculature. (**C**) Superimposed and warped image of surface vasculature and layer IV histologic sections (contrast adjusted). (**D**) Digital *in vivo* image of cortical surface and implanted multidepth electrode. (**E**) Statistical parametric mapping (SPM) analysis of total hemoglobin (Hbt) response to stimulation (hot colors and cold colors represent high and low z-scores, and black and white regions of interest (ROIs) represent pixels within 50% of the maximum and minimum z-score, respectively) confirming electrode implantation within a region of high activation.

To localize the region of somatosensory ‘barrel' cortex activated by vibrissal stimulation, a preparatory two-dimensional optical imaging spectroscopy experiment was conducted in each subject. Here, the contralateral mystacial pad was stimulated with a train of electrical pulses (1.2 mA intensity and 0.3 ms pulse width) for 2 seconds at 5 Hz. A total of 30 stimulus presentation trials of 24 seconds duration were aggregated (stimulation onset at 8 seconds, intertrial interval of 26 seconds) and averaged to create a mean trial which was subjected to the aforementioned spectral analysis. Spatiotemporal changes in Hbt were subsequently analyzed using statistical parametric mapping (SPM) in which each pixel's time series was regressed against a design matrix representing a direct current (DC) offset, ramp, and ‘boxcar' function of the same duration as the stimulation.^19^ This produced a z-score activation map where high values represented large evoked increases in Hbt, with low values conversely associated with decreases. Pixels within 50% of the maximum and minimum z-score were used to define the ‘positive' and ‘negative' regions of interest (ROIs), respectively. Positive ROIs were then coregistered with camera images of the cortical surface to guide further thinning of the skull and a small perforation in the dura mater to be made, enabling insertion of a depth electrode into the vibrissal cortex (Figures 1D and 1E). In all subjects, a 16-channel electrode, coupled to a fluidic probe loaded with 4-AP, was inserted into, and normal to, the RVC to a depth of 1,500 *μ*m. In six further animals, a second identical electrode, albeit not coupled to an infusion port, was also implanted into the left vibrissal cortex (LVC) after following the above procedure. Multichannel electrodes (16 channels with 100 *μ*m spacing, site area 177 *μ*m^2^, 1.5 to 2.7 M*Ω*s impedance, and 33 *μ*m tip width; Neuronexus Technologies, Ann Arbor, MI, USA) were coupled to a preamplifier and data acquisition device (Medusa Bioamp, TDT, Alachua, FL, USA). We found no evidence of cortical spreading depression during a 1-hour monitoring period between electrode implantation and data collection, consistent with previous reports.^9, 10, 20^

### Multidepth Electrophysiology and Current Source Density Analysis

Field potential recordings across 16 channels in each electrode were sampled at 6 kHz with 16-bit resolution and averaged over trials, with stimulus onset ‘jittered' within a 20-ms window to reduce the effects of 50 Hz mains noise. Interpretation of FPs is difficult due to the conduction properties of the extracellular medium as well as the superposition principle, in which FPs from multiple cells linearly summate and thus may not be localized within a particular cortical region. As a result, CSD analysis of laminar FP recordings was performed to resolve such inherent spatial ambiguities and transform these into a laminar distribution of current sources and sinks. This technique has been described in detail previously,^11^ and produces a characteristic superficial current source with a prominent underlying early middle-layer (‘primary') current sink in response to mystacial pad stimulation. The locus of this primary current sink (∼400 to 500 *μ*m below cortical surface) has been previously shown, using cytochrome oxidase histology, to be spatially concordant with cortical lamina IV,^21^ the target layer of primary somatosensory input from the ventral posterior thalamus. As the current sink is widely considered to reflect active, rather than passive (i.e., source related), neural mechanisms, the time series through the primary sink has been historically taken as our dependent measure of neural activity, reflecting the summed activity of excitatory postsynaptic potentials due to thalamocortical activation and subsequent intracortical processing.^20^ Comparison of this neural metric with associated hemodynamic measures to investigate neurovascular coupling is therefore warranted from both a signal processing and a neurophysiologic point-of-view. To facilitate description of neural responses to stimulation in each condition, neural data are also presented as neural profiles representing peak negative responses to each electrical pulse in the stimulation train.

### Experimental Paradigm

The stimulation protocol used to investigate neurovascular coupling consisted of 30 stimulation presentation trials with an intertrial interval of 70 seconds, each delivering a 16-second train of electrical pulses (5 Hz, 1.2 mA intensity, and 0.3 ms pulse width) to the mystacial pad after a 10-second prestimulation baseline period. These stimulation parameters have been previously shown to induce robust neural and hemodynamic responses, with maximal signal-to-noise ratio and avoidance of signal saturation in urethane anesthetized rats.^9, 16, 20, 21, 22^ Electrophysiology measures of neural activity were recorded across all 16 channels for a period of 25 seconds and beginning 4 seconds before stimulation onset in each trial. In addition, continuous neural data from one channel at a depth of 700 *μ*m were also recorded. Cortical hemodynamic measures were recorded concurrently and continuously throughout each experiment. Identification of positive and negative ROIs using SPM analysis was conducted for each hemodynamic variable in each condition, and used to derive hemodynamic time series responses to stimulation (as fractional changes from baseline unless otherwise specified).

In six subjects, an initial experimental run was conducted to obtain control neurovascular responses to stimulation of the left mystacial pad (henceforth referred to as ‘control') using the above paradigm. A short time after, and after a baseline recording of 280 seconds, 1 *μ*L of 4-AP was infused into RVC via the fluidic port in the multichannel electrode over a 5-minute period. The stimulation protocol was then repeated 400 seconds after offset of 4-AP infusion. Onset and offset timings of 4-AP-induced seizure-like discharges were evaluated by eye with reference to raw FP recordings, summed power spectrum of these in the frequency range of 0.1 to 49 Hz, and concurrent Hbt measures, with stimulation trials coinciding within ictal discharges selectively extracted and averaged for final analysis. Neurovascular responses to the aforementioned stimulation trials will be henceforward referred to as ‘intraictal'. A final experimental run, identical to the control, was then conducted 2 hours after onset of 4-AP infusion when seizure-like activity had ceased (hereafter referred to as ‘postictal' neurovascular responses to stimulation). Furthermore, in another six subjects, two control experiments were conducted with stimulation to the left (*N*=4), and right (*N*=6), mystacial pad. A further experiment was then performed in which 4-AP was infused in an identical manner to the previous one, but in the absence of any vibrissal stimulation. Finally, the control experiments were repeated postictally; the first, concerned with left mystacial pad stimulation, taking place 2 hours after commencement of 4-AP infusion, and the second, pertaining to right mystacial pad stimulation, conducted ∼2.5 hours after infusion.

### Cytochrome Oxidase Histochemistry

Following most experiments, postmortem brain tissue was prepared for histologic analysis using a modified procedure to that described by Wong-Riley and Welt,^23^ and has been described in detail previously.^24^ Briefly, subjects were perfused transcardially with saline, 4% paraformaldehyde and subsequently photographic emulsion (Jessops Ltd., Leicester, UK) to allow discrimination of surface vasculature in subsequent histologic sections. Brains were removed, the cortex of interest detached, compressed, and then sectioned into slices tangential to the surface using a cryostat, the first slice at a depth of 200 *μ*m to capture and later visualize the surface vasculature, with subsequent slices at successive 50 *μ*m depths. Slices were placed in an incubation medium in a dark room at 37°C to allow staining for cytochrome oxidase so as to distinguish barrel representations. Finally, photomicrographs of each histologic section were produced and linearly warped to each other manually (Figures 1A to 1C).

## Results

### Neural Responses to Control Stimulation of Left Mystacial Pad

**Figure 2:**
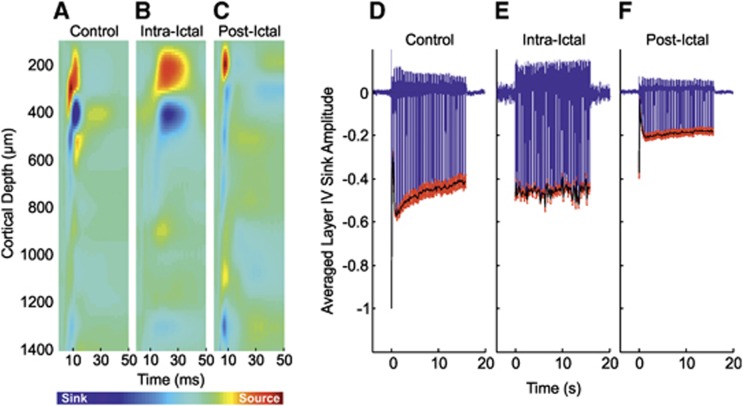
Neural responses in right vibrissal cortex (RVC) to left mystacial pad stimulation before, during, and after 4-aminopyridine (4-AP) infusion in RVC. (**A**–**C**) Representative trial-averaged current source density (CSD) analysis of laminar field potentials (FPs) in response to the first pulse of the stimulation train during ‘control', ‘intra-ictal', and ‘post-ictal' conditions, respectively. (**D**–**F**) Averaged time series derived from layer IV CSD sink (as defined in control condition) during ‘control' (*N*=10), ‘intraictal' (*N*=6), and ‘postictal' conditions (*N*=10), respectively. Black solid line represents neural profile (peak negative responses to each pulse in stimulation train). Red error bars are s.e.m.

Control stimulation of the left mystacial pad elicited typical neural responses in the contralateral RVC. The trial-averaged CSD depth profile to the first pulse of the stimulation train (Figure 2A, in a representative subject) displayed a large current source near the cortical surface (∼300 *μ*m) and underlying short-latency current sink (∼450 *μ*m, peak latency of 9±0.4 ms, *N*=10) associated with summed excitatory postsynaptic potential activity due to thalamocortical input activation and subsequent intracortical processing in layer IV.^20^ The sink subsequently propagated into more superficial layers (i.e., II/III), reflecting the activation of superficial pyramidal cells via supragranular intracortical projections and axon collaterals from layer V pyramidal cells. The trial-averaged time series through the primary layer IV sink in each subject was taken as the dependent neural metric and averaged over subjects (Figure 2D, *N*=10). Again, control responses to stimulation exhibited archetypal features, such as a robust initial response to the first pulse in the stimulation train, with gradual adaptation to later pulses, such that the amplitude at the end of stimulation was ∼40% that of the initial response.

### Hemodynamic Responses to Control Stimulation of Left Mystacial Pad

**Figure 3:**
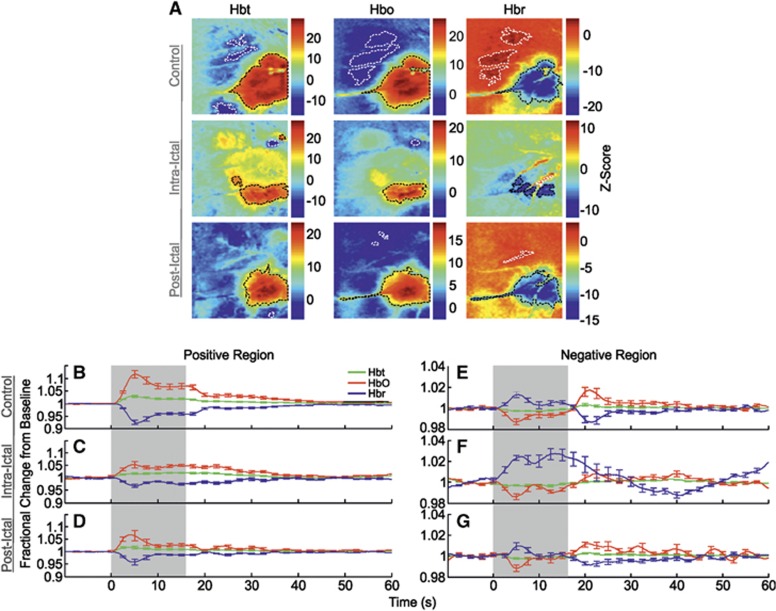
Hemodynamic responses in right vibrissal cortex (RVC) to left mystacial pad stimulation before, during, and after 4-aminopyridine (4-AP) infusion in RVC. (**A**) Statistical parametric mapping (SPM) activation maps for all hemodynamic variables during ‘control', ‘intraictal', and ‘postictal' conditions in a representative subject. Positive and negative/inverted regions of interest (ROIs) are delimited by black and white dashed lines, respectively. Hot and cold colors denote high and low z-scores. (**B**–**G**) Averaged time series of cortical hemodynamic responses from positive and negative/inverted ROIs during ‘control' (*N*=10), ‘intraictal' (*N*=6), and ‘postictal' *(N*=10) conditions, as fractional changes from baseline (Hbt, total hemoglobin; HbO, oxyhemoglobin; Hbr, deoxyhemoglobin). Shaded area indicates stimulation period. Error bars are s.e.m.

Correspondingly, control stimulation of the left mystacial pad produced stereotypical SPM activation maps of Hbt, HbO, and Hbr in RVC (Figure 3A, in a representative subject). Large increases in Hbt and HbO were observed over the entirety of the vibrissal cortex, with a robust decrease in Hbr colocalized to same general region (delimited as black dashed lines in Figure 3A). In addition to such hallmark spatial responses to stimulation, cortical areas outside the barrel field were also associated with inverted or ‘negative regions' (encircled as white dashed lines in Figure 3A) where, contrastingly, Hbt and HbO decreased, and Hbr increased. Trial-averaged hemodynamic time series, as fractional changes from baseline, for Hbt, HbO, and Hbr in both positive and negative regions were derived from ROIs and averaged over subjects (Figures 3B and 3E, *N*=10). Hemodynamic time series from the positive region (Figure 3B) exhibited ‘peak and plateau' dynamics as previously observed;^9^ the Hbt and HbO time series initially underwent a large increase in concentration that peaked after ∼5 seconds (Hbt peak maxima: 1.03±0.01 at 4.6 seconds; HbO peak maxima: 1.12±0.02 at 5.1 seconds, Table 1) and subsequently decreased to an elevated plateau that persisted for the remainder of the stimulation before a return to baseline. In contrast, the Hbr time series underwent a large decrease in concentration that reached a minimum at ∼6 seconds (Hbr peak minima: 0.93±0.01 at 5.6 seconds, Table 1) before plateauing at an increased level until stimulation offset and returning to baseline thereafter. Hemodynamic time series from negative regions (Figure 3E) also displayed characteristic dynamics as described previously.^7^ Namely, Hbt and HbO decreased (Hbt peak minima: 0.997±0.001 at 7.9 seconds; HbO peak minima: 0.987±0.003 at 4.9 seconds), and Hbr increased (Hbr peak maxima: 1.013±0.003 at 5.4 seconds) during stimulation, followed by a baseline overshoot (Hbt peak maxima: 1.003±0.001 at 20.4 seconds; HbO peak maxima: 1.017±0.005 at 21 seconds), and undershoot (Hbr peak minima: 0.987±0.003 at 21.4 seconds), respectively, before returning to baseline.

### Neurovascular Responses During 4-Aminopyridine Seizure Induction

**Figure 4:**
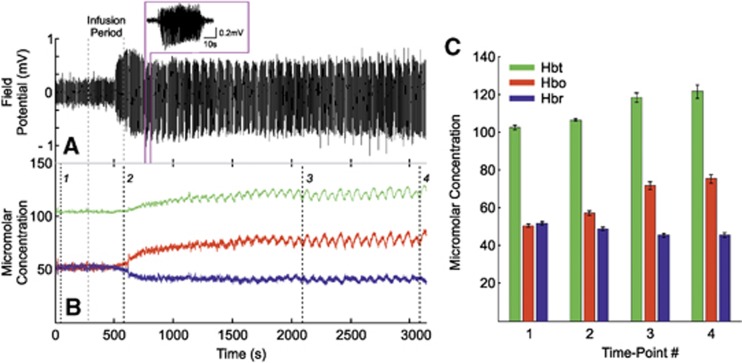
Local neural and hemodynamic baseline changes after 4-aminopyridine (4-AP) infusion in right vibrissal cortex (RVC). (**A**) Representative continuous field potential (FP) recordings made from a depth of 700 μm below cortical surface, showing evolution into spontaneous ictal-like discharges after 4-AP infusion (individual seizure shown as inset). (**B**) Corresponding Hbt, HbO, and Hbr micromolar concentration time series taken from positive regions of interest (ROIs) derived during control condition, showing marked increases in baseline Hbt and HbO, and decrease in Hbr (concomitant stimulation epochs omitted for clarity). Infusion period in both (**A**) and (**B**) indicated by gray dashed lines. (**C**) Averaged Hbt, HbO, and Hbr micromolar concentration (*N*=6) at four different time points indicated by black dashed lines in (**B**), confirming overall baseline increases in Hbt, HbO, and a decrease in Hbr after 4-AP infusion. Error bars are s.e.m. Hbt, total hemoglobin; HbO, oxyhemoglobin; Hbr, deoxyhemoglobin.

Infusion of 4-AP into the RVC induced ictal-like discharges as described previously.^12, 13^ Pronounced increases in FP activity became observable toward cessation of the infusion period and evolved into distinct spontaneous seizure-like discharges within 8 to 10 minutes after infusion, each lasting ∼30 to 80 seconds in duration, for up to 2 hours (representative example of continuous FP recordings made from a depth of 700 *μ*m is shown in Figure 4A with individual seizure selected as inset). Continuous measurements of Hbt, HbO, and Hbr micromolar concentration were also concurrently recorded (Figure 4B, from the same subject). Before the end of the infusion period, a marked increase in Hbt and HbO, and a prominent decrease in Hbr, was observed that persisted throughout the remainder of the recordings. To examine this, four time points were selected during the continuous hemodynamic recordings: (1) a baseline measure taken 60 seconds after recording onset, (2) at offset of 4-AP infusion, (3) two-thirds into the recording period, and finally (4), 60 seconds before recording offset. Hbt, HbO, and Hbr micromolar concentrations were extracted in each case and averaged over subjects (Figure 4C, *N*=6). This analysis showed a marked increase in Hbt (102.3±1.3 to 121.4±3.4 *μ*mol/L), HbO (50.5±0.7 to 75.3±2.4 *μ*mol/L), and a decrease in Hbr (51.7±0.7 to 45.5±1.2 *μ*mol/L), from baseline to the end of the recording period. Note the variations in Hbt, HbO, and Hbr during individual seizures even in the presence of such large scale concentration changes over time.

### Neural Responses to Stimulation of Left Mystacial Pad During Local Seizure Discharges

Neural responses to stimulation trials occurring during ictal discharges displayed some notable differences in comparison with control responses. The trial-averaged CSD depth profile (Figure 2B, from the same subject shown in Figure 2A) indicated the layer IV sink to be delayed (peak latency 17.5±1.5 ms, *N*=6) compared with control. Temporal broadening of the sink, which persisted during propagation of excitatory postsynaptic potential activity into supragranular regions, was also observed (Figure 2B, *N*=6). Neural responses differed from control in that the large-amplitude response to the initial stimulation pulse and subsequent adaptation was absent (Figure 2E), and instead exhibited a near steady-state amplitude throughout the stimulation period, not dissimilar to that observed at the end of control stimulation. Comparison of the summed neural profile with stimulation (solid black line in Figures 2D to 2F) revealed no significant difference between control and intraictal conditions (*P*=0.23, unpaired *t*-test).

### Hemodynamic Responses to Stimulation of Left Mystacial Pad During Local Seizure Discharges

Trial-averaged time series, as fractional changes from baseline, for Hbt, HbO, and Hbr were once again extracted from SPM-derived ROIs (Figure 3A, in a representative subject) and averaged over subjects (Figure 3C, *N*=6). In this condition, Hbt and HbO increased to a reduced amplitude peak compared with control (Hbt peak maxima: 1.02±0.01 at 13.3 seconds; HbO peak maxima: 1.05±0.01 at 4.6 seconds, Table 1) before returning to baseline similarly to control by ∼50 seconds. The Hbr time series mirrored that of HbO (Hbr peak minima: 0.97±0.01 at 5.3 seconds, Table 1). Region of interest areas of hemodynamic variables were generally reduced compared with control, although this was not found to be robustly significant (*P*>0.01 in all cases, unpaired *t*-test).

Hbt and HbO time series from negative regions (Figure 3F) were roughly comparable to those seen during control (Hbt peak minima: 0.996±0.001 at 6.1 seconds; HbO peak minima: 0.986±0.003 at 4.8 seconds), but displayed smaller and more delayed overshoots after stimulation (Hbt peak maxima: 1.003±0.001 at 38.6 seconds; HbO peak maxima: 1.008±0.004 at 22.4 seconds). The Hbr time series, however, exhibited a much larger increase (Hbr peak maxima: 1.027±0.005 at 13.5 seconds) and a more delayed undershoot (Hbr peak minima: 0.987±0.002 at 40.8 seconds). Negative regions of all variables were reduced in size, with significant decreases in Hbt and HbO response areas (*P*<0.01 in both cases, unpaired *t*-test), and not colocalized with those during the control condition. Although the mechanisms remain unclear, such differences may be due to interhemispheric inhibitory activity, thought to underpin negative hemodynamic responses,^7^ being modulated during seizures, as well as ongoing hemodynamic activation during ictal discharges (Figure 4C) occupying different cortical territories compared with baseline.

### Postictal Neural Responses to Stimulation of Left Mystacial Pad After Seizure Activity

Postictal neural responses to stimulation 2 hours after seizure induction showed similar dynamics but markedly reduced amplitudes in comparison with control. In this condition, the primary sink peaked earlier (7.9±0.2 ms) and was reduced in terms of amplitude, both at inception, and during subsequent propagation into more superficial layers (Figure 2C, from the same subject shown in Figures 2A and 2B). No significant differences between summed neural activity to postictal stimulation were found between data sets in which intraictal stimulation was conducted (*N*=6) and those in which stimulation was not delivered (*N*=4) (*P*=0.9, unpaired *t*-test). As a result, the trial-averaged time series of the layer IV primary sink was averaged over pooled subjects (Figure 2F, *N*=10). Here, as in control, the first stimulation pulse elicited the largest response with subsequent adaptation to ∼45% of this by the end of stimulation. However, though comparable to that in control, postictal responses underwent comparatively more rapid adaptation, promptly reaching an approximate steady state within a few seconds of stimulation onset. Also of note was a diminished neural response amplitude throughout the stimulation period compared with control; analysis of integrated neural activity during postictal stimulation showed there to be a 58.6% reduction compared with control and a significant decrease between control and postictal conditions (*P*=3.2 × 10^−7^, paired *t*-test). Similarly, postictal integrated neural activity was significantly reduced by 57.7% compared with the intra-ictal condition (*P*=1.7 × 10^−6^, unpaired *t*-test).

### Hemodynamic Responses to Stimulation of Left Mystacial Pad After Seizure Activity

The SPM maps of hemodynamic responses to stimulation 2 hours after seizure-induction exhibited increases in Hbt and HbO, and decreases in Hbr, that were confined to smaller areas compared with control (Figure 3A). Negative regions were likewise reduced in size and occupied different cortical localities. No significant differences in summed postictal hemodynamic responses were found between subjects in which stimulation trials were delivered during seizure activity (*N*=6) and those in which it was absent (*N*=4) (*P*=0.44, unpaired *t*-test). As such, trial-averaged hemodynamic time series obtained from SPM-derived ROIs were averaged over the combined number of subjects (Figures 3D and 3G, *N*=10). This showed a reduction in peak amplitude of all hemodynamic variables (Hbt peak maxima: 1.02±0.01 at 3.4 seconds; HbO peak maxima: 1.07±0.02 at 4 seconds; Hbr peak minima: 0.96±0.01 at 4.6 seconds, Table 1) which occurred ∼1 second earlier than that observed during control stimulation (Figure 3D). Furthermore, subsequent ‘plateaus' were diminished, resulting in a comparatively faster return to baseline. Activation regions of all variables were reduced in size compared with control, although these were not found to be significant (*P*>0.3 in all cases, paired *t*-test).

Finally, negative responses (Figure 3G) consisted of a solitary initial early peak (Hbt peak minima: 0.998±0.001 at 8.8 seconds; HbO peak minima: 0.99±0.003 at 5.1 seconds; Hbr peak maxima: 1.01±0.003 at 5.4 seconds) followed by a later overshoot (undershoot in the case of Hbr) at ∼21 seconds (Hbt peak maxima: 1.003±0.001 at 20.9 seconds; HbO peak maxima: 1.01±0.003 at 20.6 seconds; Hbr peak minima: 0.99±0.002 at 21.4 seconds) before returning to baseline. Similarly, negative regions of all variables were reduced in area compared with the control condition, but not found to be significant (*P*>0.05 in all cases, paired *t*-test).

### Neurovascular Responses to Control and Postictal Stimulation of Right Mystacial Pad

**Figure 5:**
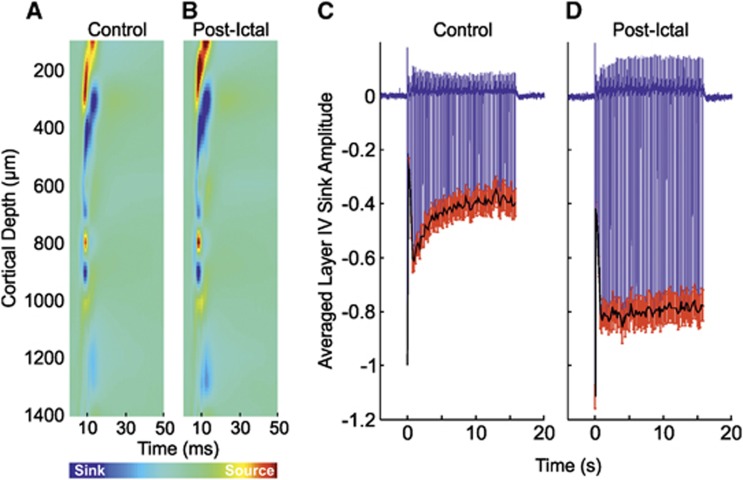
Neural responses in left vibrissal cortex (LVC) to right mystacial pad stimulation before, and after, 4-aminopyridine (4-AP) infusion in right vibrissal cortex (RVC). (**A**, **B**) Representative trial-averaged current source density (CSD) analysis of laminar field potentials (FPs) in response to the first pulse of the stimulation train during ‘control' and ‘postictal' conditions, respectively. (**C**, **D**) Averaged time series (*N*=6) derived from layer IV CSD sink (as defined in control condition) during ‘control' and ‘postictal' conditions, respectively. Black solid line represents neural profile (peak negative response to each pulse in stimulation train). Red error bars are s.e.m.

**Figure 6:**
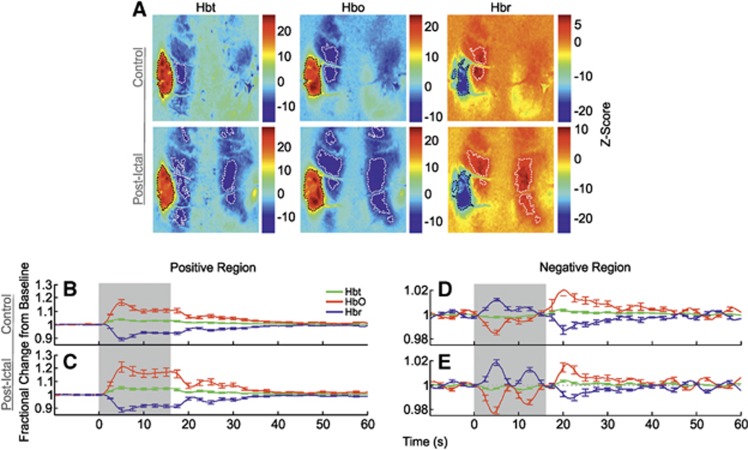
Hemodynamic responses in left vibrissal cortex (LVC) to right mystacial pad stimulation before, and after, 4-aminopyridine (4-AP) infusion in right vibrissal cortex (RVC). (**A**) Statistical parametric mapping (SPM) activation maps for all hemodynamic variables during ‘control' and ‘postictal' conditions in a representative subject. Positive and negative/inverted regions of interest (ROIs) are delimited by black and white dashed lines, respectively. Hot and cold colors denote high and low z-scores. (**B**–**E**) Averaged time series of cortical hemodynamic responses from positive and negative/inverted ROIs during ‘control' (*N*=6) and ‘postictal' *(N*=6) conditions, as fractional changes from baseline (Hbt, total hemoglobin; HbO, oxyhemoglobin; Hbr, deoxyhemoglobin). Shaded area indicates stimulation period. Error bars are s.e.m.

Neural and hemodynamic responses to control stimulation of the right mystacial pad (Figures 5A, 5C, 6A, 6B, and 6D) were, as to be expected, highly comparable to those observed during stimulation of the left mystacial pad (Figures 2A, 2D, 4A, 4B, and 4E); as such, the reader is directed to the description of the latter detailed in earlier text. Interestingly, neurovascular responses to postictal stimulation differed to those in the control condition. The primary current sink was increased in amplitude compared with control, both during initiation and subsequent proliferation into shallower cortical depths (Figure 5B), with no significant difference in peak latency between conditions observed. This was exemplified by an increased initial response in the averaged layer IV sink time series (Figure 5D, *N*=10) by ∼10% compared with control. Subsequent neural responses remained comparatively more elevated, and underwent less adaptation to ∼70% of the initial response amplitude. Summed neural activity during postictal stimulation was significantly increased by 62.8% compared with control (*P*<0.01, paired *t*-test). Resultant hemodynamic time series, as fractional changes from baseline, extracted from postictal ROIs (Figure 6C) displayed enhanced Hbt, HbO, and Hbr responses (Hbt peak maxima: 1.05±0.01 at 5 seconds; HbO peak maxima: 1.21±0.04 at 5.5 seconds; Hbr peak minima: 0.88±0.02 at 5.6 seconds, Table 1) compared with control (Hbt peak maxima: 1.04±0.01 at 4.4 seconds; HbO peak maxima: 1.16±0.02 at 4.9 seconds; Hbr peak minima: 0.89±0.01 at 5.1 seconds, Table 1). Interestingly, postictal positive ROIs became more pronounced in contralateral cortex. Both positive and negative regions of all variables were increased in size compared with control, although no significant differences in response areas were found between conditions (*P*>0.2 in all cases). Postictal hemodynamic responses in negative regions corresponded closely with those during the control condition, with the added feature of an increased ∼0.1 Hz oscillatory component, also known as vasomotion (Figure 6E).

### Epilepsy-Induced Alterations in Sensory-Evoked Neurovascular Coupling

**Figure 7:**
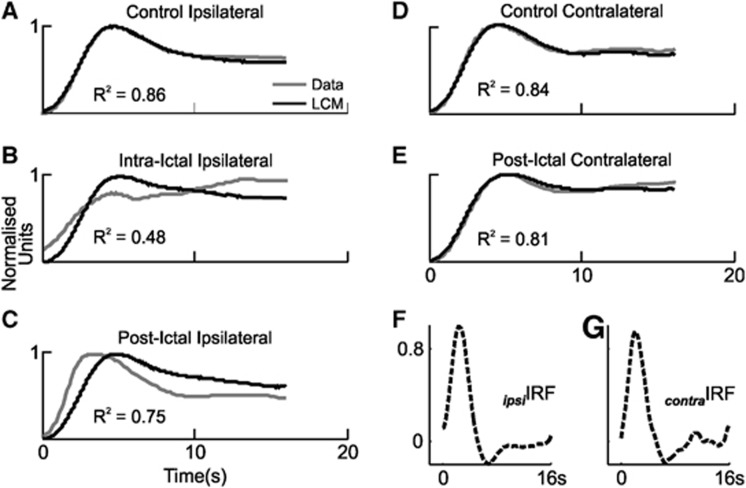
Linear convolution modeling (LCM) predictions of evoked hemodynamic (total hemoglobin, Hbt) responses. (**A**–**E**) Comparison of averaged evoked hemodynamic responses (gray) in right vibrissal cortex (LVC) with stimulation of left (right) mystacial pad during all conditions, to predictions (black) obtained through LCM and convolution of corresponding neural responses with the associated control hemodynamic impulse response function (IRF, shown in panels **F** and **G**). Coefficients of determination (*R*^2^) shown as insets. LVC, left vibrissal cortex.

We have shown that neurovascular responses to vibrissal stimulation are modified in sensory cortex in the presence of focal seizure-like discharges, and bilaterally during the postictal period. However, these findings alone are not sufficient to evaluate whether neurovascular coupling is also altered during such conditions. One method of evaluating the changes in the neurovascular coupling relationship is to deconvolve the full-wave rectified neural response time series and Hbt time series from the control condition to produce a hemodynamic impulse response function (IRF) as described previously.^8, 10, 11^ Convolution of neural time series in other conditions with this ‘control' IRF produces predictions of hemodynamic responses under the assumption that the system is linear time invariant. Failure of linear convolution modeling to faithfully predict experimentally obtained Hbt responses is therefore indicative that the neurovascular coupling relationship has undergone a departure from linearity. Thus, IRFs were derived in each control condition (i.e., ipsilateral and contralateral to SOZ, Figures 7F and 7G) and convolved with evoked neural responses in each condition to yield Hbt response predictions. Unsurprisingly, linear convolution modeling predictions in control conditions ipsilateral and contralateral to SOZ provided appropriate fits to the experimentally obtained Hbt/CBV responses (Figures 7A and 7D), as quantified by the coefficient of determination (*R*^*2*^=0.86 and 0.84, respectively). However, predictions were inadequate with regard to Hbt responses to stimulation during seizure activity (*R*^*2*^=0.48, Figure 7B), and only reasonable in the postictal condition (*R*^*2*^=0.75, Figure 7C, although note the later predicted onset). These results suggest that seizure activity modulates sensory-evoked neurovascular coupling, the subtleties of which may endure up to 2 hours after seizure induction. In contrast, postictal Hbt responses contralateral to SOZ were essentially equally well predicted as during the control condition, suggesting that neurovascular coupling is unchanged in such regions (*R*^*2*^=0.81, Figure 7E). Impulse response functions obtained through deconvolution of neural and hemodynamic responses in each condition were in keeping with these findings (data not shown). Notably, while the derived intraictal IRF was markedly attenuated compared with control and displayed a strong oscillatory component, postictal IRFs exhibited similar dynamics to control, with the exception of late oscillatory components and, ipsilateral to SOZ, a reduced latency initial peak.

## Discussion

The key findings of this study were the following: (1) sensory-evoked neurovascular coupling in the SOZ is modified during focal ictal-like discharges, (2) induction and persistence of seizure activity after injection of 4-AP produces marked increases in Hbt, HbO, and a decrease in Hbr concentration, in addition to polyphasic alterations underlying each ictal event, (3) sensory-evoked neurovascular responses are significantly reduced in vibrissal cortex ipsilateral to SOZ, and reciprocally enhanced in contralateral vibrissal cortex. These novel observations have implications for understanding sensory-evoked neurovascular coupling and dynamics in focal neocortical epilepsy, the interpretation of noninvasive functional neuroimaging data during epileptic states, and provide insights into the characteristics of the 4-AP model of neocortical seizures in the anesthetized rodent.

### Neurovascular Responses During 4-Aminopyridine-Induced Seizure Activity

We have shown there to be a divergence from linearity in the sensory-evoked neurovascular coupling relationship in the SOZ during 4-AP-induced ictal discharges. If debilitation of sensory-evoked neurovascular coupling is taken to reflect an underlying perturbation of neurovascular mechanisms, then our observation supports and extends the suggestion that neurovascular coupling in epilepsy may be altered from the normal state.^12, 13, 25^ Taken together, these findings suggest caution when interpreting perfusion-based imaging signals during ictal activity, such as increasingly popular BOLD fMRI, which traditionally assume linearity in neurovascular coupling under the framework of the general linear model.^14^ Furthermore, the observed increases in Hbt/CBV and decreases in Hbr after 4-AP infusion may also be an important consideration as most models of the BOLD signal are heavily dependent on baseline cerebral blood flow and Hbr.^26^ Accurate interpretation of perfusion-related signals is predicated on a detailed knowledge of the underpinning neurovascular coupling mechanisms, much of which has thus far been derived through examination of sensory-evoked neurovascular responses in anesthetized rodent preparations.^5, 6, 7, 9, 10, 16, 20, 21, 27^ Since the use of noninvasive functional mapping seems destined to play an increasingly important role in identification of epileptogenic foci and adjacent functional cortex,^28^ insights into neurovascular coupling during seizures, such as those presented here, are central to enabling their faithful interpretation.

Contemporaneous with local seizure activity, we observed neural responses to undergo strong adaptation and exhibit temporal broadening, which may be ascribed to the action of 4-AP that produces prolonged presynaptic depolarization leading to increases in inhibitory and, particularly, excitatory neurotransmitter release.^29, 30^ We and others have suggested that the ‘peak and plateau' response to long duration stimulation may represent two distinct neurovascular coupling components; an initial transient arterial backwards dilation followed by an increase in CBV colocalized to the region of neuronal activation and possibly of metabolic origin, respectively.^10^ The slower hemodynamic response dynamics during stimulation in the presence of ictal activity, which lacked the prominent ‘peak' component compared with control, may therefore reflect a reduced capacity for further arterial dilation in the presence of the large increases in Hbt/CBV that was observed after seizure induction.

### Modulation of Postictal Neurovascular Responses

The significant reduction in sensory-evoked neurovascular responses ∼2 hours after regional seizure induction may have several explanations. 4-AP-induced seizure activity can promote neurodegeneration through excitotoxic mechanisms mediated by *N*-methyl-D-aspartate receptor overactivation, and astrocytic edema.^29, 30, 31^ The earlier onset of postictal Hbt responses is in keeping with the premise that 4-AP-induced seizures may produce a functional lesion in the cortical layers. Specifically, damage to cortical neurons would lead to reduced evoked dilation of local microvasculature, resulting in the contributions of different layers to the Hbt/CBV signal to be weighted toward more superficial layers containing large pial vessels with faster onset times.^32^ Alternatively, the diminishment of postictal sensory neural responses may be due to transitory seizure-induced changes in neuronal function such as which may underlie the clinical phenomenon of ‘Todd's paresis', a frequent occurrence in the functional cortex after a focal epileptic event. The pathophysiology of postictal paresis is incompletely understood, but regarded to be multifactorial, involving neurotransmitter and receptor alterations, active inhibition and cerebrovascular dysfunction.^33^ That sensory-evoked neurovascular coupling was preserved would suggest that cerebrovascular function is maintained during the postictal phase, but we cannot exclude neurotransmitter and receptor changes and active inhibition as possible mechanisms underlying our results.

Interestingly, we also observed an enhancement of post-ictal sensory-evoked neural responses in vibrissal cortex contralateral to the SOZ. This might be ostensibly attributed to some form of disinhibition of commissural projections between RVC and LVC known to underpin bilateral integration of sensory responses to vibrissal stimulation.^34^ However, a recent study using focal photothrombotic model of stroke and somatosensory stimulation in rat has shown there to be significantly enhanced sensory activation in contralesional, unaffected, cortices after stroke.^35^ Such enhancements were suggested to be likely mediated by subcortical connections, rather than transcallosal projections, with disinhibition of contralesional thalamic nuclei rendering these more responsive to ascending sensory inputs.^35^

### Possible Clinical Significance

Although care should be exercised when extrapolating our findings to the human epileptic brain it is tempting to speculate on the potential implications of our results. For example, in instances where epileptogenic foci are believed to be within sensory regions, the use of appropriate stimulation paradigms and evaluation of evoked neurovascular coupling before and during seizures, and subsequent mapping of areas where it is comparatively disrupted, might provide a useful adjunct when localizing the SOZ. A caveat to this is the preponderance of BOLD fMRI paradigms in epilepsy conducted during interictal periods, rather than ictal periods, mainly due to unpredictability, noise, and safety considerations associated with the latter. Notwithstanding, interictal and ictal activity may not always be colocalized,^36^ and there has thus been considerable interest gaining in ictal electroencephalogram-fMRI studies^37^ as localization of fMRI correlates of electrophysiologic seizure events may be a more efficacious method of identifying the epileptogenic zone. However, though ictal electroencephalogram-fMRI studies have typically convolved electrophysiologic discharges with a canonical hemodynamic response function to characterize seizure-related BOLD signal changes, reservations about the reliability of this general linear model approach in pathologic conditions have led to novel data-driven methods (including independent component analysis) being recently developed.^36^

Our finding of a postictal reduction in sensory function at the site of the epileptic focus is consistent with evidence of postictal diminishment of auditory event-related potentials ipsilateral to the epileptogenic zone, which have been suggested to be of localizing value in patients with temporal lobe epilepsy.^4^ However, we could find no reports of reciprocal enhancements of sensory function in cortex contralateral to SOZ. Additional clinical research may shed further light on the possibility that sensory function is augmented in contralateral cortical regions immediately after intense recurrent focal seizures, which may provide a secondary method with which to confirm laterality of the epileptogenic zone. Our finding of preserved postictal sensory-evoked neurovascular coupling suggests that perfusion-based imaging techniques would be suitable in this regard. This may also lead to a greater understanding of the impact of seizure-induced modulation of sensory function on cognitive and behavioral deficits in focal neocortical epilepsy. Finally, the data also enlighten the interpretation of intraoperative optical imaging experiments for localizing ictal and functional cortex.^28, 38^

### Methodological Considerations

In this study, we used regional injection of 4-AP to generate focal ictal-like events in the somatosensory cortex of the urethane-anesthetized rat, which enabled the use of invasive recording techniques with high spatial and temporal resolution. This acute epilepsy model remains the only *in vivo* model capable of reliably inducing stereotypical focal neocortical seizure-like discharges in the anesthetized rodent, and has therefore found widespread use in the study of neurovascular coupling in partial onset epilepsy.^12, 13, 15^ In interpreting our data, we cannot exclude the effect of 4-AP on voltage-gated potassium channels expressed on vascular smooth muscle cells. However, the expected consequence of this would be that of vasoconstriction (and thus a reduction in CBV) in arterioles originating from the middle cerebral artery that irrigate the vibrissal cortex,^39^ which is the opposite of that described here.

Urethane provides a long-lasting and stable depth of surgical anesthesia, and has been shown to preserve excitatory (glutamate mediated) and inhibitory (GABA_A_ and GABA_B_ mediated) synaptic transmission, in contrast to many general anesthetics that are thought to enhance GABAergic and inhibit glutamatergic transmission.^40^ Neurovascular coupling is also preserved under urethane such that a single whisker deflection elicits a hemodynamic response in the rat barrel cortex.^16^ These properties have led to the common use of urethane when investigating neural-hemodynamic coupling.^7, 8, 9, 10, 11, 16, 17, 18, 20, 21, 22^ Although other studies have preferred the use of *α*-chloralose, the choice of *α*-chloralose or urethane was not found to affect the spatial-temporal pattern of the evoked hemodynamic response,^27^ nor the relationship between neural activity and BOLD fMRI responses.^22^

## Figures and Tables

**Table 1:** Peak amplitude as fractional changes from baseline of hemodynamic variables in activated regions

	*Hbt peak amplitude*	*HbO peak amplitude*	*Hbr peak amplitude*
*Left mystacial pad stimulation*			
Control	1.03±0.01	1.12±0.02	0.93±0.01
Intraictal	1.02±0.01	1.05±0.01	0.97±0.01
Postictal	1.02±0.01	1.07±0.02	0.96±0.01
			
*Right mystacial pad stimulation*			
Control	1.04±0.01	1.16±0.02	0.89±0.01
Postictal	1.05±0.01	1.21±0.04	0.88±0.02

## References

[bib1] Grant AC Interictal perceptual function in epilepsy Epilepsy Behav 2005 6 511 519 15907746 10.1016/j.yebeh.2005.03.016

[bib2] Takahashi H Straschill M The effects of focal epileptic activity on the somatosensory evoked potentials in the rat Eur Arch Psychiatry Clin Neurosci 1981 231 81 91 10.1007/BF00342832 7316737

[bib3] Masuoka LK Anderson AW Gore JC McCarthy G Spencer DD Novotny EJ Functional magnetic resonance imaging identifies abnormal visual cortical function in patients with occipital lobe epilepsy Epilepsia 1999 40 1248 1253 10487188 10.1111/j.1528-1157.1999.tb00854.x

[bib4] Abubakr A Wambacq I The localizing value of auditory event-related potentials (P300) in patients with medically intractable temporal lobe epilepsy Epilepsy Behav 2003 4 692 701 14698703 10.1016/j.yebeh.2003.09.001

[bib5] Sheth SA Nemoto M Guiou M Walker M Pouratian N Toga AW Linear and nonlinear relationships between neuronal activity, oxygen metabolism, and hemodynamic responses Neuron 2004 42 347 355 15091348 10.1016/s0896-6273(04)00221-1

[bib6] Zheng Y Pan Y Harris S Billings S Coca D Berwick J A dynamic model of neurovascular coupling: implications for blood vessel dilation and constriction Neuroimage 2010 52 1135 1147 20138217 10.1016/j.neuroimage.2010.01.102 PMC2891822

[bib7] Boorman L Kennerley AJ Johnston D Jones M Zheng Y Redgrave P Negative blood oxygen level dependence in the rat: a model for investigating the role of suppression in neurovascular coupling J Neurosci 2010 30 4285 4294 20335464 10.1523/JNEUROSCI.6063-09.2010 PMC6634501

[bib8] Bruyns-Haylett M Zheng Y Berwick J Jones M Temporal coupling between stimulus-evoked neural activity and hemodynamic responses from individual cortical columns Phys Medicine Biol 2010 55 2203 10.1088/0031-9155/55/8/006 20348608

[bib9] Kennerley AJ Harris S Bruyns-Haylett M Boorman L Zheng Y Jones M Early and late stimulus-evoked cortical hemodynamic responses provide insight into the neurogenic nature of neurovascular coupling J Cereb Blood Flow Metab 2011 32 468 480 22126914 10.1038/jcbfm.2011.163 PMC3293120

[bib10] Martindale J Berwick J Martin C Kong Y Zheng Y Mayhew J Long duration stimuli and nonlinearities in the neural–haemodynamic coupling J Cereb Blood Flow Metab 2005 25 651 661 15703699 10.1038/sj.jcbfm.9600060

[bib11] Martindale J Mayhew J Berwick J Jones M Martin C Johnston D The hemodynamic impulse response to a single neural event J Cereb Blood Flow Metab 2003 23 546 555 12771569 10.1097/01.WCB.0000058871.46954.2B

[bib12] Ma H Zhao M Schwartz TH Dynamic neurovascular coupling and uncoupling during ictal onset, propagation, and termination revealed by simultaneous *in vivo* optical imaging of neural activity and local blood volume Cereb Cortex 2012 23 885 899 22499798 10.1093/cercor/bhs079 PMC3593576

[bib13] Zhao M Ma H Suh M Schwartz TH Spatiotemporal dynamics of perfusion and oximetry during ictal discharges in the rat neocortex J Neurosci 2009 29 2814 2823 19261877 10.1523/JNEUROSCI.4667-08.2009 PMC2745405

[bib14] Worsley KJ Friston KJ Analysis of fMRI time-series revisited—again Neuroimage 1995 2 173 181 9343600 10.1006/nimg.1995.1023

[bib15] Schwartz TH Bonhoeffer T *In vivo* optical mapping of epileptic foci and surround inhibition in ferret cerebral cortex Nat Med 2001 7 1063 1067 11533712 10.1038/nm0901-1063

[bib16] Berwick J Johnston D Jones M Martindale J Martin C Kennerley A Fine detail of neurovascular coupling revealed by spatiotemporal analysis of the hemodynamic response to single whisker stimulation in rat barrel cortex J Neurophysiol 2008 99 787 798 18046008 10.1152/jn.00658.2007 PMC2652198

[bib17] Berwick J Johnston D Jones M Martindale J Redgrave P McLoughlin N Neurovascular coupling investigated with two-dimensional optical imaging spectroscopy in rat whisker barrel cortex Eur J Neurosci 2005 22 1655 1666 16197506 10.1111/j.1460-9568.2005.04347.x

[bib18] Kennerley AJ Berwick J Martindale J Johnston D Zheng Y Mayhew JE Refinement of optical imaging spectroscopy algorithms using concurrent BOLD and CBV fMRI Neuroimage 2009 47 1608 1619 19505581 10.1016/j.neuroimage.2009.05.092 PMC2782682

[bib19] Friston KJ Frith C Liddle P Frackowiak R Comparing functional (PET) images: the assessment of significant change J Cereb Blood Flow Metab 1991 11 690 699 2050758 10.1038/jcbfm.1991.122

[bib20] Harris S Jones M Zheng Y Berwick J Does neural input or processing play a greater role in the magnitude of neuroimaging signals Front Neuroenerget 2010 2 1 7 10.3389/fnene.2010.00015 PMC2927268 20740075

[bib21] Jones M Hewson-Stoate N Martindale J Redgrave P Mayhew J Nonlinear coupling of neural activity and CBF in rodent barrel cortex Neuroimage 2004 22 956 965 15193627 10.1016/j.neuroimage.2004.02.007

[bib22] Huttunen JK Gröhn O Penttonen M Coupling between simultaneously recorded BOLD response and neuronal activity in the rat somatosensory cortex Neuroimage 2008 39 775 785 17964186 10.1016/j.neuroimage.2007.06.042

[bib23] Wong-Riley M Welt C Histochemical changes in cytochrome oxidase of cortical barrels after vibrissal removal in neonatal and adult mice Proc Natl Acad Sci 1980 77 2333 2337 6246540 10.1073/pnas.77.4.2333 PMC348709

[bib24] Zheng Y Johnston D Berwick J Mayhew J Signal source separation in the analysis of neural activity in brain Neuroimage 2001 13 447 458 11170810 10.1006/nimg.2000.0705

[bib25] Voges N Blanchard S Wendling F David O Benali H Papadopoulo T Modeling of the neurovascular coupling in epileptic discharges Brain Topogr 2012 25 136 156 21706377 10.1007/s10548-011-0190-1

[bib26] Davis TL Kwong KK Weisskoff RM Rosen BR Calibrated functional MRI: mapping the dynamics of oxidative metabolism Proc Natl Acad Sci 1998 95 1834 1839 9465103 10.1073/pnas.95.4.1834 PMC19199

[bib27] Devor A Ulbert I Dunn AK Narayanan SN Jones SR Andermann ML Coupling of the cortical hemodynamic response to cortical and thalamic neuronal activity Proc Natl Acad Sci USA 2005 102 3822 3827 15734797 10.1073/pnas.0407789102 PMC550644

[bib28] Prakash N Uhlemann F Sheth SA Bookheimer S Martin N Toga AW Current trends in intraoperative optical imaging for functional brain mapping and delineation of lesions of language cortex Neuroimage 2009 47 T116 T126 18786643 10.1016/j.neuroimage.2008.07.066 PMC2782948

[bib29] Pena F Tapia R Seizures and neurodegeneration induced by 4-aminopyridine in rat hippocampus *in vivo*: role of glutamate-and GABA-mediated neurotransmission and of ion channels Neuroscience 2000 101 547 561 11113304 10.1016/s0306-4522(00)00400-0

[bib30] Pena F Tapia R Relationships among seizures, extracellular amino acid changes, and neurodegeneration induced by 4-Aminopyridine in rat hippocampus: a microdialysis and electroencephalographic study J Neurochem 1999 72 2006 2014 10217278 10.1046/j.1471-4159.1999.0722006.x

[bib31] Fabene P Weiczner R Marzola P Nicolato E Calderan L Andrioli A Structural and functional MRI following 4-aminopyridine-induced seizures: a comparative imaging and anatomical study Neurobiol Dis 2006 21 80 89 16084733 10.1016/j.nbd.2005.06.013

[bib32] Jin T Kim SG Cortical layer-dependent dynamic blood oxygenation, cerebral blood flow and cerebral blood volume responses during visual stimulation Neuroimage 2008 43 1 9 18655837 10.1016/j.neuroimage.2008.06.029 PMC2579763

[bib33] Fisher RS Schachter SC The postictal state: a neglected entity in the management of epilepsy Epilepsy Behav 2000 1 52 59 12609127 10.1006/ebeh.2000.0023

[bib34] Shuler MG Krupa DJ Nicolelis MAL Bilateral integration of whisker information in the primary somatosensory cortex of rats J Neurosci 2001 21 5251 5261 11438600 10.1523/JNEUROSCI.21-14-05251.2001 PMC6762838

[bib35] Mohajerani MH Aminoltejari K Murphy TH Targeted mini-strokes produce changes in interhemispheric sensory signal processing that are indicative of disinhibition within minutes Proc Natl Acad Sci 2011 108 E183 E191 21576480 10.1073/pnas.1101914108 PMC3107306

[bib36] LeVan P Tyvaert L Moeller F Gotman J Independent component analysis reveals dynamic ictal BOLD responses in EEG-fMRI data from focal epilepsy patients Neuroimage 2010 49 366 19647798 10.1016/j.neuroimage.2009.07.064 PMC3779215

[bib37] Salek-Haddadi A Merschhemke M Lemieux L Fish DR Simultaneous EEG-correlated ictal fMRI Neuroimage 2002 16 32 40 11969315 10.1006/nimg.2002.1073

[bib38] Zhao M Suh M Ma H Perry C Geneslaw A Schwartz TH Focal increases in perfusion and decreases in hemoglobin oxygenation precede seizure onset in spontaneous human epilepsy Epilepsia 2007 48 2059 2067 17666071 10.1111/j.1528-1167.2007.01229.x

[bib39] Horiuchi T Dietrich HH Tsugane S Dacey RG Role of potassium channels in regulation of brain arteriolar tone comparison of cerebrum versus brain stem Stroke 2001 32 218 224 11136940 10.1161/01.str.32.1.218

[bib40] Sceniak MP MacIver MB Cellular actions of urethane on rat visual cortical neurons *in vitro* J Neurophysiol 2006 95 3865 3874 16510775 10.1152/jn.01196.2005

